# Editorial: The embodied brain: computational mechanisms of integrated sensorimotor interactions with a dynamic environment, volume II

**DOI:** 10.3389/fncom.2023.1203717

**Published:** 2023-05-16

**Authors:** Florian Röhrbein, Mario Senden

**Affiliations:** ^1^Faculty of Computer Science, Technische Universität Chemnitz, Chemnitz, Germany; ^2^Faculty of Psychology and Neuroscience, Maastricht University, Maastricht, Netherlands

**Keywords:** embodiment, sensorimotor, dynamic environment, neurorobotics, closed-loop

Embodiment and situatedness play an increasingly important role in our understanding of the brain. In recent years, advances in theoretical neuroscience, robotics and applied mathematics have provided researchers with new tools to study the perception-action cycle in naturalistic environments. The second volume of The Embodied Brain: Computational Mechanisms of Integrated Sensorimotor Interactions with a Dynamic Environment brings together experts from these and other fields to explore the latest developments in the study of embodied sensorimotor systems. With their diverse expertise, the contributors to this volume provide important insights into the mechanisms underlying embodied cognition and sensorimotor integration.

The paper by Scleidorovich et al. studies the impact of multi-scale spatial navigation in large and cluttered environments, which is a challenging task for animals as well as humans. Evidence shows that the mammalian brain has evolved to use multiple concurrent spatial navigation scales and to use spatial memory to improve path planning. How these scales are used and what advantages they provide are largely unknown. In four experiments the authors study how the hippocampus of rats may distribute place cell fields in an environment to support efficient learning of optimized goal-oriented trajectories.

In an ANN architecture Scholz et al. investigate a possible implementation of active inference, which formalizes how agents choose actions based on sensory information to maintain homeostasis. They propose that the information about an agent's environment can guide and alleviate the costs of goal-directed planning via active inference and demonstrate that it enables their architecture to avoid obstacles and regions of uncertainty and to generalize learned associations to similar environments.

The active inference framework (AIF) proposed by Yang et al. is a neuroscience-based framework that can produce human-like behavior through reward-based learning. For a better understanding of visually guided action and active inference they developed an agent that selects from a set of discrete actions in order to perform the well-known task of interception. They demonstrated the use of AIF as a plausible model of anticipatory behavior for visual-motor tasks.

The paper by Steinmetz et al. introduces a self-tuning mechanism for capturing rapid adaptation to changing visual stimuli by a population of neurons. The authors show how neural tuning curve parameters can be continually updated to optimally encode a time-varying distribution of stimulus values. They conclude that dynamic efficient sensory encoding offers a plausible approach for capturing adaptation to varying visual environments.

Taken together, these papers from labs in the US and Germany demonstrate the breadth and importance of embodied sensorimotor systems and the potential for interdisciplinary research to provide new insights into the complex relationship between action, perception and the environment. By bringing together experts from different fields, we hope to further advance our understanding of the brain and its interactions with the environment, and to develop new tools and models to support future research in this area.

## Author contributions

Both authors listed have made a substantial, direct, and intellectual contribution to the work and approved it for publication.

